# I Got Rhythm and Executive Function, Memory, and More: The Automated Test of Embodied Cognition (ATEC)

**DOI:** 10.3390/brainsci15030299

**Published:** 2025-03-12

**Authors:** Morris D. Bell, Yarani Gonzalez, Andrea J. Weinstein, David Ciosek, Yan Wang, Gihyun Yoon

**Affiliations:** 1 Department of Psychiatry, Yale University School of Medicine, New Haven, CT 06510, USA; yarani.gonzalez@yale.edu (Y.G.); andrea.weinstein@yale.edu (A.J.W.); yan.wang.yw476@yale.edu (Y.W.); gihyun.yoon@yale.edu (G.Y.); 2 VA Connecticut Healthcare System, West Haven, CT 06516, USA; david.ciosek@va.gov

**Keywords:** rhythm, embodied cognition, ATEC, neurocognition and movement, story memory, visual memory, executive functions

## Abstract

**Background**: The Automated Test of Embodied Cognition (ATEC) is a new system for measuring cognition in action that uses cognitively demanding physical tasks and motion capture technology. Rhythm is one of the domains assessed by the ATEC across a number of tasks and is a domain for which there is no broadly accepted neurocognitive measure. **Method**: Rhythm was assessed in a sample of 104 participants that included those at risk of cognitive decline and community controls. At-risk participants were also administered standard measures of executive functioning (EF), verbal list-learning, story memory, visual memory, and pre-morbid IQ. **Results**: The ATEC Rhythm Domain was found in the factor analysis with Varimax rotation to be loaded distinctly on the EF factor. ATEC Rhythm was significantly correlated with EF neurocognitive measures, and, in a Chi-square analysis, significantly differentiated the community control participants from those at risk for cognitive decline. ATEC Rhythm was significantly correlated with story memory and visual memory but not verbal list-learning. Age was negatively correlated with ATEC Rhythm, and women performed slightly better than men. ATEC Rhythm was also significantly correlated with the years of education and an estimate of pre-morbid IQ. **Discussion**: ATEC Rhythm was found to have discriminant and concurrent validity with EF measures and was significantly correlated with measures of story memory and visual memory, but not verbal list-learning. We speculate on rhythm’s relationship to story narrative and visual sequencing, and on rhythm’s relationship to cognitive reserve, as represented by education and the pre-morbid IQ estimate. **Conclusions**: The ATEC is a promising new measure that provides a systematic assessment of rhythm as a domain of embodied cognition. It may be useful in studies of neurodevelopment and neurocognitive decline, and it may be especially useful in assessing the effects of interventions that use physical activities, including dance and music therapies.

## 1. Introduction

The Automated Test of Embodied Cognition [1,2] is a new assessment instrument that uses cognitively demanding physical tasks (like an exercise video) to evaluate cognitive functions in action. Performance is digitally recorded using a conventional webcam and scored on eight cognitive domains, one of which is rhythm. Rhythmic activity was scored across a number of tasks, including “March to the Beat”, an embodied memory task called “Map Sense” measuring spatial memory and navigation skills, a Go/No-Go task called “Red Light/Green Light”, and a self-regulation task called “Cross Your Body”. These will be described in Section 2.

Rhythm was built into these tasks based on the literature (briefly reviewed below) that recognizes that staying in rhythm (that is, responding to patterns of structured temporal sequences) requires complex higher cortical processes as well as coordinated movement, and that rhythmic training has been found to enhance cognitive development and to benefit those in cognitive decline. By integrating rhythmic demands into these tasks, the ATEC offers a broad measurement of rhythmic performance within the context of other cognitive demands. This broad construct of rhythm includes auditory rhythm perception, temporal motor coordination, general motor planning, attention, working memory, response inhibition, and cognitive flexibility.

Neuroscience of Rhythm: Rhythmic sensory input results in the activation of the bilateral frontal, cingulate, parietal, prefrontal, and temporal cortices, which is to say, the vast majority of the neocortex [3]. Interestingly, the processing of tempo, or rhythmic speed, uniquely engages the somatosensory and premotor cortex [4]. The auditory perception of rhythm also activates and causes a specific response in the basal ganglia, the cluster of brain structures responsible for movement control and inhibition [4,5]. The simultaneous engagement of frontal, prefrontal, premotor, and movement areas suggests that multiple levels of cognition are required for the perception of rhythm, and that the execution of rhythmic movement cannot be separated from higher-level cognitive function.

Research on the cerebellum and rhythmic motor behavior highlights the cerebellum’s essential role in motor coordination, error correction, and temporal processing during rhythm perception and execution. The cerebellum ensures the precise timing of actions, enabling individuals to synchronize their movements with external rhythm cues [6]. Along with the basal ganglia and prefrontal cortex, the cerebellum also regulates the initiation and execution of movements [7,8]. The prefrontal cortex plays an important role in the cognitive control necessary for rhythmic responses by orchestrating thought and action in accord with internal goals [9]. Thus, the cerebellum collaborates with other brain regions in producing complex rhythmic behaviors.

Neurodevelopment and Rhythm: The processes associated with rhythmic movement begin to develop in infancy, with infants engaging in rhythmic movement when exposed to music and adjusting their movement speed to reflect the auditory tempo [10]. This skill develops into beat synchronization, which can be used to predict the language learning abilities of preschoolers, specifically reading [11]. Children with better rhythm entrainment skills also have stronger language skills, including verbal memory [12]. The breadth of rhythmic ability’s predictive power extends to general cognitive function and attention as children grow older, and can be used to accurately predict performance in school [13,14], including developmental dyslexia [15,16]. The connection between rhythm and cognitive ability has been further supported by experimental studies, which have found that auditory musical training significantly improves the performance on measures of verbal intelligence and plasticity [17,18]. Motor rhythm training also improved language-based cognitive task performance, but not other aspects of executive function, like attention, inhibition, or working memory [19].

Rhythm as Therapy: Rhythm training has been shown to improve outcomes for individuals with autism spectrum disorder (ASD), though the exact cause of its success is not determined. Their social deficits are partially the result of a poor ability to predict and respond to social cues, which manifest as poor ability to time responses [20]. The repetitive nature of rhythm lends itself to a predictable type of social training and has been shown to improve social and communication skills in individuals with ASD [21,22,23,24,25,26].

The benefit of rhythm training can be applied to neurodegenerative disorders, most notably Parkinson’s Disease (PD). The presence of a regular acoustic stimulus causes improvements in gait stability and coordination, which is thought to be the result of synchronization to a predicted beat, which overrides the deficient internal timer [27]. The improvements extend into complex coordination between upper and lower body movement, allowing for more complicated sequences of movement [27]. Improvements are not limited to physical function, but cognitive function as well, primarily in flexibility, semantic word retrieval, and working memory [27]. It is possible that the spatial proximity of the semantic recovery region and the temporal lobe causes some crossover that improves retrieval with rhythmic auditory stimulation [17]. 

The benefit of rhythm training is also reflected in enhancing brain plasticity and improving cognitive reserve. Cognitive reserve refers to the brain’s ability to maintain cognitive function despite aging or neurological damage. A higher cognitive reserve is associated with stronger rhythmic abilities due to more resilient and adaptable brain networks [28].

Such functional crossover would allow for the reasonable prediction that neurological disorders involving other brain regions that activate during rhythm perception can also be treated with rhythm therapy. Huntington’s disease causes the progressive degeneration of the basal ganglia [29,30], adding to impairments in movement. Finally, therapies involving rhythmic auditory stimulation-based movement training, music therapy, and dance, which have been found to be beneficial for PD and Huntington’s disease, may also be useful for other cognitive disorders, including traumatic brain injury (TBI) and dementia. These therapies have been shown to improve emotional adjustment and executive function in individuals with a variety of cognitive impairments [31,32,33,34].

Measurement of Rhythm: Among the most prominent attempts to assess rhythm was the Seashore measures of musical talent, which measured the ability to perceive rhythm and maintain rhythm in the absence of a sensory stimulus [35]. A study using this battery tested for motor rhythm, the ability to sustain a regular rhythm with a regular bodily movement, and uncovered a variable involved in the execution of motor rhythm: basic rhythm. The Seashore Rhythm Test [36] was subsequently added to the Halstead–Reitan neurocognitive test battery [37]. The task has thirty pairs of rhythmic patterns presented in a series of ten items. The first series contains patterns of five notes, the second with patterns of six notes, and the third with patterns of seven notes. The purpose is to indicate whether the two patterns are the same or different. No body movement is involved in this task, and later research concluded that the task made no unique diagnostic contribution within the context of a neuropsychological test battery [37,38]. 

Although rhythm involving body movement has been shown to involve higher cognitive processes, and to have potential clinical and therapeutic value, there is currently no systematic assessment system for determining individual differences, or to relate those differences to specific cognitive processes or disorders. The ATEC provides the first opportunity to do so.

In this report, we hypothesize that ATEC Rhythm can significantly discriminate between participants at risk for cognitive decline and community control participants. We also test the hypotheses that ATEC Rhythm is significantly related to measures of executive function and memory, and that ATEC Rhythm is also associated with cognitive reserve in those at risk for cognitive decline.

## 2. Materials and Methods

### 2.1. Participants

We are currently conducting a National Institute on Alcohol Abuse and Alcoholism (NIAAA)-funded study (RO1AA029075) of cognitive training and donepezil in Alcohol Use Disorder (AUD), for which the ATEC is one of the cognitive assessments. We are also conducting another study on the ATEC itself, which includes older participants at risk for cognitive decline due to AUD, mild traumatic brain injury (mTBI), or other early-stage neurological disorders. Community controls with no known cognitive risk were previously administered using the ATEC during the test development and are included in this report [39]. Because of different protocols, the sample sizes for the correlations vary according to whether the measure was used in one protocol or more than one protocol. Inclusion/exclusion criteria for the AUD study included the presence of a current diagnosis of AUD, as determined using the Mini International Neuropsychiatric Interview (M.I.N.I) [40], and the absence of a psychotic, neurological, sensory, or orthopedic disorders that would interfere with the performance of the ATEC. For the second study, the inclusion criterion was any diagnosis that might put a participant at risk of cognitive decline, and the same exclusion criteria were used as in the AUD study. The sample (N = 104) was 61.3% male, with a mean age of 49.65 (16.76); 22.6% were US Veterans; Mean Years of Education = 15.62 (2.99); 96.1% were right-handed. The sample was 67.3% White, 21.2% African American or Black, 2.9% Hispanic, 3.8% Asian, and 4.8% Other. For those in the NIAAA study (n = 35), the mean of lifetime use of alcohol to intoxication was 247.86 months (194.14), and their Wechsler Test of Adult Reading (WTAR) [41] mean score, used as an estimate of the pre-morbid IQ, was 111.00 (12.68).

### 2.2. Measures: Automated Test of Embodied Cognition

The ATEC consists of complex tasks related to executive function in motion (e.g., the “Cross Your Body” task, described below), and a number of simpler movement tasks (e.g., Timed Up and Go, tandem walk, hand pronation–supination, and finger tapping) commonly used in neurological exams and utilized in the Movement Disorder Society-Unified Parkinson’s Disease Rating Scale (MDS-UPDRS) [42] (see the scoring pamphlet in the Supplementary Material). Video administration automated the tasks and increased the fidelity of the assessment, while motion capture and artificial intelligence/machine learning (AI/ML) scoring algorithms decreased the measurement error. The system includes a software interface linked to the demonstration video and the motion capture system.

#### 2.2.1. ATEC Novel Tasks and Rhythm

To test the higher cognitive processes related to executive functions, novel physical tasks were added to the standard neurological tasks. One of the three core tasks with higher cognitive demand is an attention and response inhibition task called “Red Light/Green Light/Yellow Light”. It is similar to computerized continuous performance tests (CPTs) [43] that assess sustained attention and response inhibition but is more complex and requires rhythmic upper body movement in response to commands. The participant is asked to raise and pass a juggling ball from one hand to the other in rhythm to the words “Green Light”, to move the ball up and down to the words “Yellow Light”, and to not pass the ball when the participant hears “Red Light”. The task is subsequently repeated at a faster pace. The participant is then presented with the same task but using a sequence of pictures of red, green, and yellow traffic lights as visual cues, rather than the spoken cues, thus allowing for comparison between sensory modalities. The auditory or visual prompts are presented first at a slower tempo and then again at a faster tempo, thus increasing the cognitive load by demanding more rapid processing and movement. The task is scored for accuracy, response inhibition (not moving on Red Light), and rhythm (raising the ball on the words Green or Yellow and dropping the ball into the opposite hand on the word Light, on the beat).

The second novel task is the “Cross Your Body” task. In beat to a tune, participants are instructed to touch their ears alternately with the opposite hand (left hand to right ear; right hand to left ear) three times, and then their knees three times (Lyrics: “Cross your body, touch your ears, ears, ears; Cross your body, touch your knees, knees, knees”). Then, the participants are instructed to touch their knees when the word “ears” is heard and touch their ears when the word “knee” is heard. The same procedure then replaces ears and knees with hips and shoulders. In the final round, all 4 commands are given, and the person must remember to touch their knees when “ears” is heard, to touch their ears when “knees” is heard, to touch their hips when “shoulders” is heard, and to touch their shoulders when “hips” is heard. This task requires sustained attention, working memory, response inhibition, cognitive flexibility, and self-regulation. Crossing the midline increases the sensitivity for the detection of subtle brain compromise and increases the cognitive load. The task is scored for accuracy and rhythm (touching the body part on the beat).

The third task is an embodied memory task called “Map Sense”. It requires the participant to remember 3-, 4-, and then 5-step movements across a 3 × 3 grid. The steps are sequentially displayed on a map on a screen, and then the participant must remember the sequence and move across the grid on the floor in rhythm to a simple tune. There are three trials for each map. The participant is tested again 20 min later without the maps being shown to measure the delayed recall. The task is scored for accuracy and rhythm (stepping into the squares on the beat). A list of the ATEC tasks is shown in Table 1, along with the cognitive demands that they represent.

The fourth task used to measure rhythm only is a Marching task. The participant is asked to march in place, raising one knee and swinging the opposite arm, and then doing the same with the other knee and arm, to a 4/4 count for 18 s. There are 20 beats at a slower pace and 32 beats at a faster pace. Marching to the beat means that their foot hits the floor on each count. There is a demonstration of the task on screen and a practice round to make sure the participant understands the directions.

Expert scoring created raw scores at the item level (e.g., the number of marching steps on the beat and accurate ball passes), and these raw scores were then categorized, summed across individual tasks, and then converted to a 6-point scale (0–5) so that each component score of each task (e.g., accuracy and rhythm) had equal value. The Rhythm Domain has a maximum of 38 points, with cut-off scores for conversion into a 0-to-5-point scale (i.e., 33–38 points = 5, 26–32 = 4, 19–25 = 3, 13–18 = 2, 6–12 = 1, and 0–5 = 0; see the scoring pamphlet in the Supplementary Material). For this report, the data were based on expert scoring (inter-rater agreement, ICC r = 0.935; *p* < 0.001) [1] because the AI/ML algorithms are still in development. The expert scoring served as the “ground truth” for the algorithm development. At the time of scoring, the hypotheses for this study had not yet been formulated, so no unintended bias would have likely influenced the findings of this report.

#### 2.2.2. Neurocognitive Tests

Executive function (EF) was measured using the Neuropsychological Assessment Battery (NAB) Mazes [44] and the Wisconsin Card Sorting Test (WCST) [45]. Visual memory was measured using the Brief Visual Memory Test (BVMT) [46] and BVMT delayed recall. Verbal learning and memory was assessed using the Hopkins Verbal Learning Test (HVLT) [47] with delayed recall, and story memory was evaluated using Logical Memory I [48] and Logical Memory II for delayed recall. Pre-morbid intelligence was estimated using the Wechsler Test of Adult Reading [41].

### 2.3. Procedures

Participants were administered the ATEC and other measures in accordance with approved protocols by the IRB at the VA Connecticut Healthcare System (VACHS), and included US Veteran and non-Veteran samples. Participants signed a written informed consent document and a HIPAA authorization. All of the testing was performed in private offices and in the ATEC Lab, a dedicated space for ATEC evaluations that is large enough for a participant to perform the Timed Up and Go task, involving standing up from a seated position and walking 10 feet to a floor marker, and then walking back. It takes approximately 45 min for the ATEC tasks and approximately 1 hour for the neurocognitive assessments. Participants were invited to take breaks as necessary to minimize fatigue.

### 2.4. Analysis

All of the analyses were performed using SPSS v. 29 0.2.0. The ATEC Domain scores were categorized according to the scoring pamphlet from No Impairment (score = 5) to Very Severe Impairment (score = 0; see the scoring pamphlet). The cut-offs for the categories were based on clinical practice and the cut-offs for the MDS-UPDRS, which was a model for the ATEC. A normative sample is not available at this time. A principal component analysis with Varimax rotation was used to determine the factor structure of the ATEC because, with moderate levels of intercorrelation among the domains, orthogonality would ensure that each factor was conceptually and statistically distinct. Correlations were performed using Pearson correlations for the parametric data and Spearman correlations for the non-parametric data. All of the tests were two-tailed with an alpha of 0.05 for significance. The Skew and Kurtosis for the Rhythm Domain was found to be within acceptable limits for the parametric analyses, as were all of the neurocognitive assessments. Categorical analyses were performed using the Pearson Chi-square test and the Fisher Exact Test.

## 3. Results

### 3.1. Factor Analysis (Table 2; N = 104)

The factor analysis yielded a three-factor solution, with the Rotation Sums of Squared Loadings explaining 44.048% of the variance for the first factor, 17.480% for the second factor, and 14.006% for the third factor, for a cumulative factor of 75.534%. As shown in Table 2, the Rotated Component Matrix showed that Rhythm was most highly weighted on the Executive Function factor, along with Self-Regulation and Response Inhibition, and made no significant contribution to the Embodied Memory and Motor Speed factors.

**Table 2:** Rotated component matrix.

	Component		
	1	2	3
BALANCE	0.730	−0.067	0.408
WORKING MEMORY	0.710	0.475	−0.063
RESPONSE INHIBITION	0.831	0.151	0.096
SELF REGULATION	0.899	0.191	0.070
RHYTHM	0.810	0.159	0.054
ATTENTION	0.563	0.547	−0.168
EMBODIED MEMORY RECALL	0.083	0.881	0.166
MOTOR SPEED	0.093	0.096	0.937

Extraction method: Principal Component Analysis. Rotation method: Varimax with Kaiser Normalization.

### 3.2. Distribution of Rhythm Scores (Figure 1; N = 104)

**Figure 1:**
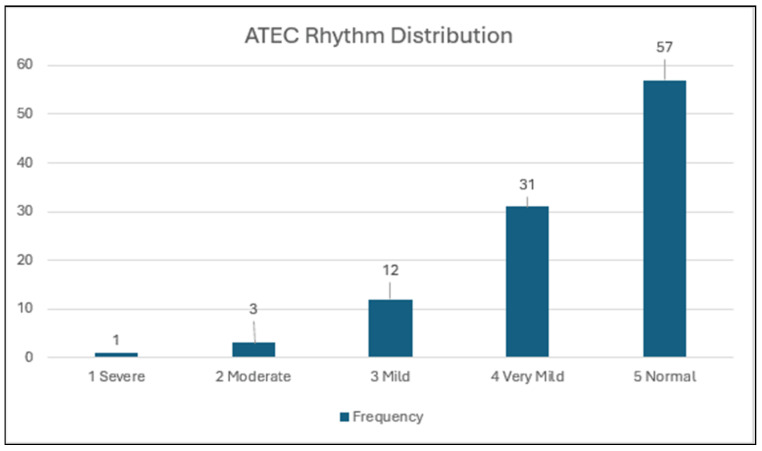
Distribution of ATEC Rhythm scores.

Rhythm scores were within the normal range (score = 5) for 57 participants (54.8%), while 16 participants (15.4%) were in the mild-to-severe range (3 to 1). No participant received a score of zero, which would be in the Very Severe Range, so Figure 1 does not include it. For the entire sample, the mean score = 4.35 (0.868).

### 3.3. Comparison of AUD/At-Risk Sample with Community Controls (Table 3; N = 90)

To compare the AUD/at-risk sample with the community controls, we chose to exclude participants who were greater than 70 years old. We did so because the ATEC does not yet have age-related norms, and we were concerned that including our oldest participants might confuse the comparison, since almost by definition they are at risk of cognitive decline. This reduced the sample size by 14 participants, and most of those excluded were from the community control sample. The mean age for the AUD/at-risk sample = 48.23 (13.44), and the community controls = 43.21 (16.30), which was not a significant difference (*p* = 0.129). Gender was also not significantly different between the groups (n = 90, Chi-square = 0.076 and df = 1; *p* = 0.783). The cross-tabulation of the Rhythm Impaired (scores of 1 to 4) or Unimpaired (score of 5) yielded a significant Pearson Chi-Square = 5.214 (1), *p* = 0.022, and a Fisher’s Exact Test score, *p* = 0.035.

**Table 3:** Cross-tabulation of the AUD/at-risk and community controls by Rhythm Impaired or Unimpaired.

		Impaired	Unimpaired	Total
AUD/At-Risk	Count	29	33	62
	% within AUD/At-Risk	46.8%	53.2%	100%
Cty Control	Count	6	22	28
	% within Cty Controls	21.4%	78.6%	100%

Chi Sq = 5.214 and df = 1, *p* = 0.022; Fisher’s Exact Test, *p* = 0.035; Phi = 0.241.

### 3.4. Correlations with Neurocognitive Assessments

#### 3.4.1. Executive Function Measures and ATEC Rhythm Scores

Rhythm correlated significantly with the Mazes age-adjusted t-score (n = 72, r = 0.322; *p* < 0.01). The WCST Percent Conceptual Response age-adjusted t-score (n = 34, r = 0.454; *p* < 0.01) and WCST Categories Correct raw score (n = 34, r = 0.511, *p* < 0.01) were also significantly related to Rhythm.

#### 3.4.2. Memory Measures and ATEC Rhythm Scores

Verbal learning and memory was assessed using the HVLT with delayed recall. Story memory was assessed using Logical Memory I and delayed story memory using Logical Memory II. Visual memory was assessed using the BVMT with delayed recall. All of the memory scores were age-adjusted t-scores. Rhythm was significantly correlated with Logical Memory I (n = 34, r = 0.462; *p* < 0.01) and Logical Memory II (n = 34, r = 0.415; *p* = 0.015), and with the BVMT total recall (n = 54, r = 0.494; *p* < 0.001) and BVMT delayed recall (n = 54, r = 0.613; *p* < 0.001). However, Rhythm was not significantly correlated with the HVLT total recall (n = 35, r = 0.02; *p* = 0.909) or with the HVLT delayed recall (n = 34, r = 0.240; *p* = 0.171).

#### 3.4.3. Cognitive Reserve and ATEC Rhythm

To determine the relationship between Rhythm and measures of cognitive reserve, correlations were performed with years of education (data available for the entire sample) and the WTAR, a reading test, widely regarded as a measure of pre-morbid intelligence (for our AUD sample only). Rhythm was significantly correlated with years of education (N = 104, r = 0.359; *p* < 0.001) and with the WTAR age-adjusted standard scores (n = 35, r = 0.466; *p* < 0.01).

### 3.5. Age, Gender, and Rhythm

The age range for the entire sample was from 19 to 89 years (mean = 49.65 (16.76)) and was significantly negatively correlated with Rhythm (N = 104, r = −0.475; *p* < 0.001). The entire sample was 61.5% male, and females were significantly better performers than males on Rhythm (r = 0.219; *p* = 0.05), although the effect size was small.

## 4. Discussion

This is the first report on the relationship between ATEC Rhythm to EF and memory function in adults. Our factor analysis revealed that the Rhythm factor loading was almost entirely with the first factor containing EF domains, suggesting that ATEC Rhythm is strongly associated with EF performance. This finding is in line with our literature review, which related rhythm to the complex neural networks connecting the cerebellum with other brain structures, including the prefrontal cortex [49]. It hardly showed any relationship to the Motor Speed or Embodied Memory factors. Rhythm is very sensitive to added cognitive load. When added cognitive effort was needed for accuracy on complex tasks like Red Light/Green Light/Yellow Light or Cross Your Body (tasks scored for Response Inhibition and Self-Regulation), staying in rhythm became more difficult.

ATEC Rhythm also showed discriminant validity in significantly differentiating AUD/at-risk individuals from the community control participants. ATEC Rhythm further demonstrated its association with EF by being significantly correlated with the WCST Conceptual Response and WCST Categories Correct, as well as with NAB Mazes. These findings support the relevance of ATEC Rhythm to our understanding of how EF is an emergent property of the brain, requiring inputs from neurocircuits that may include cerebellar processes, as well as those of higher cognitive process involving the temporal lobe, anterior cingulate, and prefrontal cortex.

Strong relationships were found between ATEC Rhythm and the story memory tasks Logical Memory I and II (Delayed Recall), probably because both motor rhythm and story memory require organization skills, temporal sequencing, attention abilities, and predictive processing [50,51]. Also, strong relationships were found between ATEC Rhythm and the visual memory tasks BVMT and BVMT delayed recall, probably because both motor rhythm and visual memory require visual–motor integration, the retrieval of visual–spatial information, attention, and temporal organization skills [52,53]. Thus, these memory tasks and ATEC Rhythm may share additional neurobiological mechanisms, such as the role of the cerebellum in rhythmic timing and sequence learning.

However, there was no significant relationship with the verbal list-learning tasks, HVLT or HVLT delayed recall. Verbal learning may involve more diverse cognitive processes, including linguistic processing and the strategy of categorization, compared to story memory or visual memory in relation to motor rhythm [54,55]. 

We further speculate that ATEC Rhythm is engaging neurocircuits related to timing and sequence. Story memory (or episodic memory) involves a sequential narrative, with details embedded in a story that occurs over time, with a beginning, a middle, and an end. Rhythm has culturally been a part of teaching sequences and telling stories because they help narrative recall. After all, rhythmic tunes are used routinely to teach children new information (e.g., the ABCs song), and singers can recall all of the details of a story with complex lyrics, particularly in ballads. Homer’s *Iliad* and *Odyssey* are lengthy poetic narratives that were performed as song, and many pre-literate societies used epic poetic songs to preserve their myths and histories. 

And why would a visual memory task relate to neurocognitive process captured by ATEC Rhythm? The BVMT not only requires the respondent to remember the geometric designs, but (unlike the HVLT) they must be remembered in their order on the page. We speculate that finding a strong relationship between ATEC Rhythm and BVMT’s visual memory and its sequential demands may relate to how moving in sequence (even in imagination) aids memory. Like actors who describe that it is easier to remember their lines after the scenes have been blocked on stage [56], movement helps to provide a sequence to the story that aids memory. It may also relate to a long-recognized memory technique called the Memory Palace, which uses the method of mentally traveling through a series of rooms in which items to be remembered have been placed so that the person can recall the information sequentially. It is an ancient technique (called the Method of Loci) and improves declarative memory (like verbal list-learning) by converting it into an imagined spatial sequence with imagined motion. Using this method [57] is well recognized as a useful technique for successful recall and has been found to be related to spatial memory and episodic memory [58]. 

Finally, we found, as expected, that ATEC Rhythm in adults has a negative correlation with age, so that older people scored lower than younger people, and there is also a small effect for gender, with women doing significantly better than men. We also found that years of education and an estimate of pre-morbid IQ were associated with better ATEC Rhythm scores. These are two measures of cognitive reserve and suggest some association between rhythmic capacity and cognitive reserve.

### Limitations

The ATEC is an automated assessment with high fidelity and is a readily transferable technology with the potential for broad dissemination. However, to date, we have not gathered sufficient data to create age- and gender-related norms. Although we did not find age and gender disparities in our group comparison (AUD/at-risk vs. community controls), it will be important to repeat this analysis when the ATEC scores are normed. We also recognize that the comparison between at-risk groups and community controls will benefit from samples that are larger and of a more equal size. We also explored the relationship of ATEC Rhythm to conventional measures of EF and memory, and this too will bear repeating when the ATEC is normed. Additionally, this study used a convenience sample comprised of people with many sources of potential cognitive compromise. In the future, we expect to report on ATEC studies with more specific diagnostic groups. We may find that ATEC Rhythm is especially relevant to early-stage Parkinson’s disease, Multiple Sclerosis, or Alzheimer’s disease, but less so for other disorders.

While we believe that ATEC Rhythm may offer a new method for understanding neurocognitive processes related to EF and memory, we do not claim that these explanations for the relationship between ATEC Rhythm and story memory and visual memory are the only possible interpretations, and we regard these explanations as generative speculation. We hope that other researchers will attempt to repeat our findings and/or use other methods to further explore the role that rhythm may play in memory. We are hopeful that ATEC Rhythm will be a useful measure for the scientific exploration of the impact of rhythm-related therapeutic interventions (e.g., music therapy and dance therapy) for neurodevelopmental disorders and for cognitive decline. At the same time, we recognize that understanding the neurobiological mechanisms of our Rhythm scores must await further research. As our population ages, disorders causing cognitive decline are becoming more common, and the discovery of better therapeutics is ever more urgent. Our hope is that the ATEC may be useful in recording the functional impact of these approaches to develop personalized treatments and improve outcomes.

## 5. Conclusions

The ATEC is a promising new measure that provides a systematic assessment of rhythm as a domain of embodied cognition. It may be useful in studies of neurodevelopmental and neurocognitive decline and may be especially useful in assessing the effects of interventions that use physical activities, including dance and music therapies. ATEC Rhythm showed discriminant validity between a cognitively at-risk sample and community controls and concurrent validity with EF measures, and some, but not all, types of immediate and delayed recall. Our finding that ATEC Rhythm had significant relationships with story memory and visual recall, but not with verbal list-learning, suggests that further research is warranted to explore these differences.

## 6. Patents

The ATEC is the intellectual property of the VA, Yale University, and the University of Texas at Arlington. International and national patent protection is in process.

## Figures and Tables

**Table 1:** ATEC tasks, cognitive demands, and scoring categories.

Type	Tasks	Domains
Gross Motor and Gait, Proprioception, Vestibular ^1^	Timed Up and Go, Dual Attention, Tandem Gait, Romberg, Standing on One Foot (L, R)	Balance and Gait
Rhythmic Movement ^2^	Marching Slow and Fast	Rhythm
Embodied Memory ^2^	3-, 4-, 5-step maps	Working Memory/Rhythm
Embodied Delayed Recall ^2^	20-min Delayed Recall	Delayed Recall
Bilateral Coordination ^1^	Ball Pass to the Beat Slow and Fast	Coordination/Rhythm
Response Inhibition ^2^	Red Light/Green Light/Yellow Light Slow and Fast/Auditory and Visual	Response Inhibition, Rhythm, Attention
Bilateral Coordination and Self-regulation ^2^	Cross Your Body	Self-Regulation, Rhythm, Working Memory
Rapid Sequential Movements ^1^	Foot Tap, Foot Stomp, Fist Open and Close, Hand Pronate/Supinate, Finger Tap (L, R)	Motor Speed, Fluidity

^1^ MDS-UPDRS; ^2^ Higher Cognitive Function.

## Data Availability

Data collection is ongoing and has not been archived yet. The data presented in this manuscript are available by contacting the corresponding author.

## Supplementary Materials

The following supporting information can be downloaded at: https://www.mdpi.com/article/10.3390/brainsci15030299/s1, Figure S1: ATEC scoring pamphlet.

## Abbreviations

The following abbreviations are used in this manuscript:

ATEC	Automated Test of Embodied Cognition
AUD	Alcohol Use Disorder
EF	Executive Function
SUD	Substance Use Disorder
MoCA	Montreal Cognitive Assessment
NIAAA	National Institute on Alcohol Abuse and Alcoholism
M.I.N.I.	Mini International Neuropsychiatric Interview
WTAR	Wechsler Test of Adult Reading
MDS-UPDRS	Movement Disorder Society-Unified Parkinson’s Disease Rating Scale
AI	Artificial Intelligence
ML	Machine Learning
IVA	Integrated Visual and Auditory-2 Continuous Performance test
NAB	Neuropsychological Assessment Battery
WCST	Wisconsin Card Sorting Test
BVMT	Brief Visual Memory Test
HVLT	Hopkins Verbal Learning Test
VACHS	VA Connecticut Healthcare System
