# Supplementary material for: I Got Rhythm and Executive Function, Memory, and More: The Automated Test of Embodied Cognition (ATEC)
Source: Brain Sci. 2025 Mar 12;15(3):299. doi: 10.3390/brainsci15030299 (PMC11940590; doi:10.3390/brainsci15030299)
Supplement: Supplementary file 1 [file brainsci-15-00299-s001.zip › brainsci-3492520-supplementary.pdf]

Rater:______________________ Date:__________ Subject ID:________________

AUTOMATED TEST of EMBODIED COGNITION (ATEC)

Morris D. Bell, Ph.D.

A. GROSS MOTOR—GAIT AND BALANCE

Item	RAW	CONVERTED	SCORING CRITERIA
1. Timed-Up & Go			Steps > 20 = 0, 20-16=1, <16 = 2
2. Dual Attention			Steps > 20 = 0, 20-16=1, <16 = 2
3. Tandem Gait Forward			Steps correct: <4=0, 4-6 = 1, 7-8 = 2
4. Stand - Arms Outstretched			Seconds: <5 = 0, 5 - 8 = 1, 9 - 10 = 2
5. Stand on One Foot—Right			Seconds: <5 = 0, 5 - 8 = 1, 9 - 10 = 2
6. Stand on One Foot—Left			Seconds: <5 = 0, 5 - 8 = 1, 9 - 10 = 2
A.7. Counting Backwards 93,86,79,72,65			Numbers Correct 0=0, 1=1, 2=2, 3=>3

A.1 TOTAL GAIT/BALANCE SCORE (ITEM 1 + ITEM2 + ITEM 3)

SCORE 0 - 6 ☐

A.2 TOTAL BALANCE SCORE (ITEM 4 + ITEM 5 + ITEM 6)

SCORE 0 - 6 ☐

B. RHYTHMIC MOVEMENT

**Raw Score = total number of steps in rhythm (step on the beat)*

Item	RAW	CONVERTED	Scoring
1. March to the Beat—Slow			Beats correct: 0-8= 0, 9-16 = 1, 17-20 = 2
2. March to the Beat—Fast			Beats correct: 0-16 = 0, 17-28= 1, 29-32 = 2

B.1 TOTAL RHYTHMIC MOVEMENT SCORE

SCORE 0– 4 ☐

Rater:_____________________________________ Date:________________ Subject ID:____________________________

C. Visuospatial Memory

		ACCURACY RAW	ACCURACY CONVERTED
1. MapSense3 Trial 1	0-1=0, 2=1, 3=2		
2. MapSense3 Trial 2	0-1=0, 2=1, 3=2		
3. MapSense3 Trial 3	0-1=0, 2=1, 3=2		
4. MapSense4 Trial 1	0-1=0, 2-3=1, 4=2		
5. MapSense4 Trial 2	0-1=0, 2-3=1, 4=2		
6. MapSense4 Trial 3	0-1=0, 2-3=1, 4=2		
7. MapSense5 Trial 1	0-2=0, 3-4=1, 5=2		
8. MapSense5 Trial 2	0-2=0, 3-4=1, 5=2		
9. MapSense5 Trial 3	0-2=0, 3-4=1, 5=2		

RHYTHM/COORDINATION

		1
3	2	

	RAW	CONVERTED
0-1=0, 2=1, 3=2		
0-1=0, 2=1, 3=2		

1		
	2	
	3	4

	RAW	CONVERTED
0-1=0, 2-3=1, 4=2		
0-1=0, 2-3=1, 4=2		

	1	
	2	3
5	4	

	RAW	CONVERTED
0-2=0, 3-4=1, 5=2		
**0-2=0, 3-4=1, 5=2**		

C.1 TOTAL VISUOSPATIAL MEMORY ACCURACY SCORE

SCORE 0 - 18 [ ]

C.2 TOTAL VISUOSPATIAL MEMORY RHYTHM/ COORDINATION SCORE

SCORE 0 - 12 [ ]

D. BILATERAL COORDINATION

**each pass get up two rhythm points; one for each beat*

	ACCURACY RAW	ACCURACY CONVERTED	RHYTHM RAW	RHYTHM CONVERTED	
1. Bi-manual Ball Pass—Slow					Passes correct: 0 - 4 = 0, 5– 6 = 1, 7– 8 = 2
2. Bi-manual Ball Pass—Fast					Rhythm: 0 - 8 = 0, 9– 12 = 1, 13– 16 = 2

D.1 TOTAL BILATERAL COORDINATION

ACCURACY SCORE 0– 4 [ ]

D.2 TOTAL BILATERAL COORDINATION

RHYTHM SCORE 0– 4 [ ]

Rater:______________________________________ Date:_______________ Subject ID:___________________________

E. AUDITORY UPPER BODY ACCURACY, RHYTHM AND RESPONSE INHIBITION

**0: moves during both beats, 1: moves only on first, 2: still*

Green Lights correct: 0 - 5= 0, 6– 8= 1, 9– 10= 2	Red Lights correct: 0 - 6 = 0, 7-10= 1, 11-12= 2	Green Lights Rhythm: 0 - 10 = 0, 11-16= 1, 17- 20= 2

Bi-manual Ball Pass Red/Green Light	GREEN LIGHTS CORRECT RAW	GREEN LIGHTS CORRECT CONVERTED	RED LIGHTS CORRECT RAW	RED LIGHTS CORRECT CONVERTED	GREEN RHYTHM CORRECT RAW	GREEN RHYTHM CORRECT CONVERTED
1. Auditory—Slow	☐	☐	☐	☐	☐	☐
2. Auditory —Fast	☐	☐	☐	☐	☐	☐

Green/Yellow Lights correct: 0 - 6= 0, 7– 10= 1, 11– 12= 2	Red Lights correct: 0 - 4 = 0, 5-6= 1, 7-8= 2	Green/Yellow Lights Rhythm: 0 - 12 = 0, 13-20= 1, 21-24= 2

Ball Pass Red/Green/Yellow Light	GREEN/YELLOW LIGHTS CORRECT		RED LIGHTS CORRECT		G/Y RHYTHM CORRECT	
3. Auditory—Slow	☐	☐	☐	☐	☐	☐
4. Auditory —Fast	☐	☐	☐	☐	☐	☐

E.1 TOTAL AUDITORY ACCURACY

SCORE 0 - 8 ☐

E.2 TOTAL AUDITORY RESPONSE INHIBITION

SCORE 0 - 8 ☐

E.3 TOTAL AUDITORY RHYTHM

SCORE 0 - 8 ☐

E.4 TOTAL G/Y LIGHT ACCURACY

SCORE 0 - 4 ☐

E.5 TOTAL RED LIGHT ACCURACY

SCORE 0 - 4 ☐

F. VISUAL UPPER BODY ACCURACY, RHYTHM AND RESPONSE INHIBITION

*** No beats (rhythm)*

0: movement during the whole video

1: self-correct (move and then keep still)

2: no movement — still

Green/Yellow Lights correct: 0 - 6= 0, 7– 10= 1, 11– 12= 2	Red Lights correct: 0 - 4 = 0, 5-6= 1, 7-8= 2

Bag Pass Red/Green/Yellow Light	GREEN/YELLOW LIGHTS CORRECT		RED LIGHTS CORRECT	
1. Visual—Slow	☐	☐	☐	☐
2. Visual —Fast	☐	☐	☐	☐

F.1 TOTAL VISUAL ACCURACY

SCORE 0 - 4 ☐

F.2 TOTAL VISUAL RESPONSE INHIBITION

SCORE 0 - 4 ☐

Rater:______________________________ Date:______________ Subject ID:________________________

G. BI-LATERAL COORDINATION AND SELF-REGULATION

**Accuracy Raw Score = total number of body parts touched at least once*

**Rhythm/Coordination Raw Score = total number of times correctly touching body part with the opposite hand all three times, accurately keeping the beat for all three touches. Starting late and rushing to catch the beat is not correct*

		Accuracy RAW SCORE	Accuracy CONVERTED		Rhythm/Coordination RAW SCORE	Rhythm/Coordination CONVERTED
1. Trial 1—Ears, Shoulders, Hips and Knees **If Converted Accuracy score = 0, go to H.*	0-1=0, 2-3=1, 4=2			0-6=0, 7-10=1, 11-12=2		
2. Trial 2—Opposite Ears and Knees **If Converted Accuracy score = 0, go to H.*	0-4=0, 5-7=1, 8=2			0-12=0, 13-20=1, 21-24=2		
3. Trial 3—Opposite Hips and Shoulders **If Converted Accuracy score = 0, go to H.*	0-4=0, 5-7=1, 8=2			0-12=0, 13-20=1, 21-24=2		
4. Trial 4—Opposite Ears, Knees, Hips and Shoulders	0-6=0, 7-10=1, 11-12=2			0-18=0, 19-32=1, 33-36=2		

G.1 TOTAL ACCURACY SCORE

SCORE 0 - 8 ☐

G.2 TOTAL RHYTHM/COORDINATION SCORE 0 - 8 ☐

G.3 WORKING MEMORY ACCURACY SCORE (#2—#4)

SCORE 0 - 6 ☐

H. Visuospatial Memory Recall

		ACCURACY RAW	ACCURACY CONVERTED		RHYTHM/COORDINATION RAW	RHYTHM/COORDINATION CONVERTED
1. MapSense3 Recall	0-1=0, 2=1, 3=2			0-1=0, 2=1, 3=2		
2. MapSense4 Recall	0-1=0, 2-3=1, 4=2			0-1=0, 2-3=1, 4=2		
3. MapSense5 Recall	0-2=0, 3-4=1, 5=2			0-2=0, 3-4=1, 5=2		

MapSense3:

		1
3	2	

MapSense4:

1		
	2	
	3	4

MapSense5:

	1	
	2	3
5	4	

H.1 TOTAL VISUOSPATIAL MEMORY RECALL ACCURACY SCORE

SCORE 0 - 6 ☐

H.2 TOTAL VISUOSPATIAL MEMORY RECALL RHYTHM/COORDINATION SCORE

SCORE 0 - 6 ☐

I. RAPID/SEQUENTIAL MOVEMENTS SCORING CRITERIA

1. FOOT TAP -Rapidity Raw score = total number of correct foot taps in 10 seconds.
0-9 =0; 10-19 = 1; >20 = 2

2. FOOT STOMP- Rapidity Raw score = total number of correct foot stomp movements in 10 seconds.
0-9 =0; 10-19 = 1; >20 = 2

3. FIST OPEN CLOSE -Rapidity Raw score = total number of correct fist opening times in 10 seconds.
0-9 =0; 10-19 = 1; >20 = 2

4. HAND PRONATE/SUPINATE -Rapidity Raw score = total number of correct Pronate/Supinate movements in 10 seconds. 0-5 =0; 6-9 = 1; >10 = 2

5. FINGER TAP -Rapidity Raw score = total number of correct Finger taps in 10 seconds.
0-15 =0; 16-29 = 1; >30 = 2

****Fluidity Score ALL TASKS** — Smoothness and accuracy of motion (freezes up or other movements intrude).

Stops and doesn't restart or switches to another movement and doesn't change back to the correct movement = 0.

Stops but restarts, has intrusive movements but returns to correct movements or has mirror movements = 1.

Continuous correct movement = 2.

Rater:______________________ Date:____________ Subject ID:____________________

H. RAPID/SEQUENTIAL MOVEMENTS

	RAPIDITY RAW SCORE	RAPIDITY CONVERTED SCORE		FLUIDITY CONVERTED SCORE
FOOT TAP—RIGHT FOOT (10 SEC)				
FOOT TAP—LEFT FOOT (10 SEC)				
FOOT STOMP—RIGHT FOOT (10 SEC)				
FOOT STOMP—LEFT FOOT (10 SEC)				
FIST OPEN CLOSE —RIGHT HAND (10 SEC)				
FIST OPEN CLOSE —LEFT HAND (10 SEC)				
HAND PRONATE/SUPINATE—RIGHT HAND (10 SEC)				
HAND PRONATE/SUPINATE—LEFT HAND (10 SEC)				
FINGER TAP—RIGHT HAND (10 SEC)				
FINGER TAP—LEFT HAND (10 SEC)				
TOTAL SCORE		I.1 TOTAL RAPIDITY SCORE		I.2 TOTAL FLUIDITY SCORE

I.1 TOTAL RAPIDITY SCORE

I.2 TOTAL FLUIDITY SCORE

Rater:____ Date:________________ Subject ID:_____________________________

SUMMARY OF SCORES

BALANCE

Gait/Balance Total (A.1) [ ] + Balance Total (A.2) [ ] = Balance Total [ ]

0-1 = 0; 2-3 = 1, 4-5 = 2; 6-8 = 3; 9-10 = 4; 11-12 = 5

BALANCE CONVERTED SCORE [ ]

WORKING MEMORY

Yellow Light Accuracy Total (E.1+F.1) [ ] + Cross Body Oppo-sites (G.3) [ ] + MapSense (C.1) [ ] + Counting Backwards (A.7) [ ] = Working Memory Total [ ]

0-9 = 0; 10-16 = 1; 17-24 = 2; 25-30 = 3; 31-35 = 4; 36-39 = 5

WORKING MEMORY CONVERTED SCORE [ ]

RESPONSE INHIBITION

Bag Pass Response Inhibition Total (E.2+F.2) [ ] + Cross Body Rhythm Total (G.2) [ ] = Response Inhibition Total [ ]

0-3 = 0; 4-6 = 1, 7-10 = 2; 11-14 = 3; 15-17 = 4; 18-20= 5

RESPONSE INHIBITION CONVERTED SCORE [ ]

SELF-REGULATION

Cross Body Accuracy (G.1) [ ] + Cross Body Rhythm Total (G.2) [ ] = Self-Regulation Total [ ]

0-1 = 0; 2-3 = 1, 4-8 = 2; 9-12 = 3; 13-14 = 4; 15-16 = 5

SELF-REGULATION CONVERTED SCORE [ ]

Rhythm/Coordination

Upper Body Rhythm Total (D.2+E.3) [ ] + Whole Body Rhythm Total (G.2) [ ] + MapSense (C.2) [ ] + MapSense Recall Rhthym/Coordination (H.2) [ ] = Rhythm/ Coordination Total [ ]

0-5 = 0; 6-12 = 1; 13-18 = 2; 19-25 = 3; 26-32 = 4; 33-38 = 5

RHYTHM/COORDINATION CONVERTED SCORE [ ]

Rater:______________________________________ Date:_______________ Subject ID:____________________________

ATTENTION

Bag Pass Accuracy Total (D.1+E.1+F.1) + Cross Body Accuracy Total (G.1) + MapSense (C.1) = Attention Total

0-5 = 0; 6-13 = 1; 14-20 = 2; 21-30 = 3; 31-37 = 4; 38-42 = 5

ATTENTION CONVERTED SCORE

MOTOR SPEED

Rapidity Total (I.1) + Fluidity Total (I.2) = Motor Speed Total

0-5 = 0; 6-13 = 1; 14-20 = 2; 21-30 = 3; 31-36 = 4; 37-40 = 5

MOTOR SPEED CONVERTED SCORE

VISUAL MEMORY RECALL

Visual Memory Recall Accuracy (H.1) = Visual Memory Recall Total

0= 0; 1= 1; 2 = 2; 3-4 = 3; 5 = 4; 6 = 5

VISUAL MEMORY CONVERTED SCORE

Rater:_______________________ Date:_____________ Subject ID:____________________

AMC TOTAL SCORE

BALANCE CONVERTED SCORE ☐

+

WORKING MEMORY CONVERTED SCORE ☐

+

RESPONSE INHBITION CONVERTED SCORE ☐

+

SELF-REGULATION CONVERTED SCORE ☐

+

RHYTHM/COORDINATION CONVERTED SCORE ☐

+

ATTENTION CONVERTED SCORE ☐

+

VISUAL MEMORY RECALL CONVERTED SCORE ☐

+

MOTOR SPEED CONVERTED SCORE ☐

=

AMC TOTAL SCORE ☐

Performance Categories

**Very Severely Impaired**	**0—15**	☐
**Severely Impaired**	**16—18**	☐
**Moderately Severely Impaired**	**19—22**	☐
**Moderately Impaired**	**23—27**	☐
**Mildly Impaired**	**28—32**	☐
**Minor Difficulties**	**33—36**	☐
**No Difficulties**	**37—40**	☐

Rater:____ Date:________________ Subject ID:_____________________________

SUPPLEMENTARY SCORES

Discrepancy - Right / Left

Right	Left
A.5	A.6
H.1	H.2
H.3	H.4
H.5	H.6
H.7	H.8
H.9	H.10
Total =	Total =

☐ − ☐ = ☐

Discrepancy - Auditory / Visual

Auditory	Visual
E.4	F.1
E.5	F.2
Total =	Total =

☐ − ☐ = ☐

Discrepancy

Timed Up & Go / Dual Attention

☐ − ☐ = ☐

Discrepancy - Accuracy / Response Inhibition

Accuracy	Response Inhibition
E.1	E.2
F.1	F.2
Total =	Total =

☐ − ☐ = ☐
